# Photocatalytic Methylene Blue Degradation of Electrospun Ti–Zn Complex Oxide Nanofibers

**DOI:** 10.3390/nano10071311

**Published:** 2020-07-04

**Authors:** Wan-Tae Kim, Kyeong-Han Na, Dong-Cheol Park, Wan-Hee Yang, Won-Youl Choi

**Affiliations:** 1 Department of Advanced Materials Engineering, Gangneung-Wonju National University, Gangneung, Gangwon 25457, Korea; dktkzz1@naver.com (W.-T.K.); nag0717@naver.com (K.-H.N.); 2 WITH M-TECH Co., Ltd., Suwon, Gyeonggi 16229, Korea; cufe2000@naver.com (D.-C.P.); dangchan74@empas.com (W.-H.Y.); 3 Research Institute for Dental Engineering, Gangneung-Wonju National University, Gangneung, Gangwon 25457, Korea

**Keywords:** TiO_2_, ZnO, complex oxide, electrospinning, methylene blue degradation

## Abstract

Photocatalysts are the most important technology in air pollution removal and the detoxification of organic materials. Doping and complexation are among the most used methods to improve the efficiency of photocatalysts. Titanium dioxide and zinc oxide nanomaterials are widely used materials for photocatalysts and the degradation of toxic materials. Their mixed structure can be fabricated by many methods and the structure affects their properties. Nanofibers are efficient materials for photocatalysts due to their vertically formed structure, which improves the charge separation of photoelectrons. We fabricated them by an electrospinning process. A precursor consisting of titanium 4-isopropoxide, zinc acetate dihydrate and polyvinylpyrrolidone was used as a spinning solution for a mixed structure of titanium dioxide and zinc oxide with different molar ratios. They were then calcined, crystallized by heat treatment and analyzed by thermogravimetric-differential thermal analysis (TG-DTA), X-ray diffractometer (XRD), field emission scanning electron microscope (FE-SEM) and energy-dispersive spectroscope (EDS). After annealing, the average diameters of the Ti–Zn complex oxide nanofibers were 237.6–278.6 nm with different salt ratios, and multiple crystalline structures were observed, namely TiO_2_, ZnO, ZnTiO_3_ and Zn_2_TiO_4_. We observed the photocatalytic performance of the samples and compared them according to the photodegradation of methylene blue. The methylene blue concentration decreased to 0.008–0.650 after three hours, compared to an initial concentration of 1, with different metal oxide structures.

## 1. Introduction

Titanium dioxide has excellent chemical, electrical and optical properties, as well as chemical stability. For that reason, it is used in many different fields of study and industry, in photocatalysts, dye-sensitized solar cells, perovskite sensitized solar cells, metal oxide semiconductor gas sensors, dental implants, interferometric sensors, photonic crystals and other applications [1,2,3,4,5,6,7,8,9,10,11,12]. It is a very useful material and is commonly used for photocatalysts due to its photoactivity, high stability, low cost and safety for the environment and humans [13,14,15]. Photocatalysts are the most important technology for air pollution removal and the detoxification of organic materials. When an electron is stimulated with light energy equal to or greater than the bandgap of TiO_2_, photocatalytic reactions begin [6]. As the photo-excited electrons are transferred from the valence band to the conduction band, electron-hole pairs are produced and a powerful oxidation reaction occurs. To increase the photo-activity and efficiency of photocatalysts, researchers have fabricated and reported many nanostructures of TiO_2_, such as nanoparticles, nanofibers and nanotubes [1,5,6,7,9,10,11,12,15,16,17,18,19,20,21]. Their nanostructures were fabricated by the sol-gel method, hydrothermal treatment, anodic oxidation and electrospinning [1,3,4,5,6,7,8,9,10,11,12,13,14,15,16,18,19,20,22,23]. Electrospinning is an efficient practical technique that is low cost and has high efficiency, and many studies have reported producing these various nanofibers. In electrospinning, the precursor solution flows at a constant rate through a pump in such a way as to create a continuous nanofiber, and then electrodes are connected to the inflowing electrospinning solution and other electrodes are connected to the appliance plate. At this time, if a high voltage is applied, it is emitted in a conical shape by surface tension at the end of the electrospinning solution [21,24,25,26,27]. The charge is subsequently stored in the electrospinning solution, and the mutual repulsion causes the cone to be radiatively stretched to a jet when the surface tension of the electrospinning solution is exceeded. In the radiation-stretched electrospinning solution, volatilization of the solvent occurs before it collects in the plate, which may result in disorderly arranged nanofibers in the plate. Doping and complexation are among the most used methods to improve the efficiency of photocatalysts. Many other materials were co-doped and mixed as impurities and composites to introduce additional states in the TiO_2_ bandgap [6]. With fewer energy transitions required, these impurity levels cause visible light absorption. TiO_2_ band gap impurity levels are induced by substituting metal ions for Ti^4+^ closest to the conduction band. Zinc oxide is also widely used as a photocatalyst [28,29,30,31,32]. Nanostructured ZnO has frequently been fabricated, and it has been used in composites with TiO_2_ for photocatalysts. Many ZnO–TiO_2_ composite structures have been reported [6,18,19,33,34,35,36].

To investigate the photocatalytic performance of Ti–Zn complex oxide nanofibers, we fabricated Ti–Zn complex oxide nanofibers by electrospinning and used them as photocatalysts for methylene blue degradation with different molar ratios of Ti and Zn. The electrospinning precursors were composed of titanium 4-isopropoxide (TTIP), zinc acetate dihydrate (ZnAc), polyvinylpyrrolidone (PVP), acetylacetone (ACAC), anhydrous ethyl alcohol (EtOH) and deionized water. Their thermogravimetric analysis (TGA) curves and differential thermal analysis (DTA) curves were observed by thermogravimetric-differential thermal analysis (TG-DTA) in an air atmosphere. After heat treatment and crystallization, their crystal structures were analyzed by X-ray diffractometer (XRD). Microstructure changes were measured and compared before and after heat treatment by a field emission scanning electron microscope (FE-SEM) and the composition was analyzed by energy-dispersive spectroscope (EDS). Their photocatalytic activity was also observed and compared by ultraviolet–visible (UV-Vis) spectroscopy.

## 2. Materials and Methods

### 2.1. Materials

ACAC and TTIP were purchased from Junsei Co., Ltd. (Tokyo, Japan). PVP (molecular weight 1,300,000) was purchased from Alfa Aesar Korea Co., Ltd. (Incheon, Korea). EtOH was purchased from Samchun Chemical Co., Ltd. (Seoul, Korea). ZnAc was purchased from Daejung Chemicals & Metals Co., Ltd. (Gyeonggi, Korea).

### 2.2. Fabrication of Ti–Zn Complex Oxide Nanofibers by an Electrospinning Process

**Figure 1:**
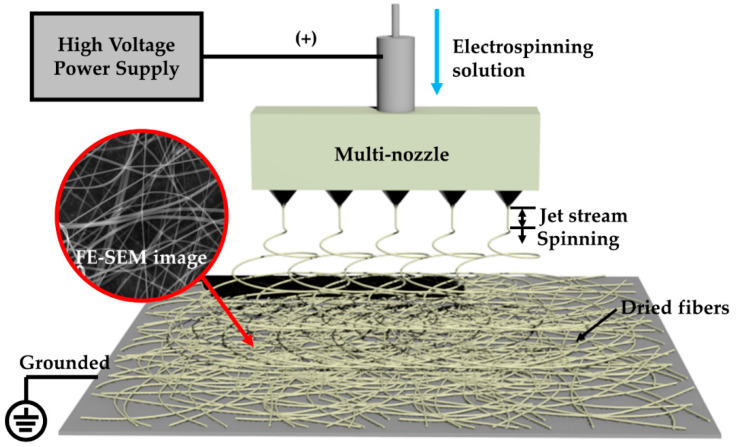
Schematic diagram of the multi-nozzle electrospinning process.

Ti–Zn complex oxide nanofibers were prepared by a multi-nozzle electrospinning process. Figure 1 shows a schematic diagram of the multi-nozzle electrospinning process. The fibrous structure was fabricated at a high voltage by high potential and volatilization of the solvent. The electrospinning solution was prepared with 5 wt% titanium and zinc salt, 5 wt% ACAC and 10 wt% PVP in a 16:64 mixture of deionized water and EtOH. TTIP and ZnAc were used for salts. The titanium and zinc salts were composed of different molar ratios according to the zinc content, and they were labelled TZ1, TZ2, TZ3, TZ4 and TZ5. Table 1 shows the molar ratios. After solution preparation, the samples were loaded into a plastic syringe and connected to a five-hole multi-nozzle adaptor. The spinning nozzle diameter was about 0.34 mm (23 gauge). The distance between the needle and the collector was 25 cm, and a high voltage of 22 kV was applied to the spinning solution by a direct current power supply. The as-spun nanofibers were collected on grounded aluminum foil.

### 2.3. Microstructural, Thermal and Crystalline Characterization

The thermal behavior of the as-spun Ti–Zn complex oxide nanofibers was analyzed by TG-DTA (STA 409, NETZSCH Korea Co., Ltd., Paju, Korea) from room temperature to 800 °C at a rate of 5 °C/min in 30 mL/min of air atmosphere, and the annealing temperature was determined. The samples were annealed for 5 h at 600 °C in an electric furnace. After heat treatment and crystallization, their crystal structures were analyzed by XRD (AXS-D8, Bruker Korea Co., Ltd., Gyeonggi, Korea, which was radiated by Cu Kα). Microstructure changes with different ratios of Ti and Zn salt were measured and compared before and after heat treatment by FE-SEM (Inspect F, FEI Korea Co., Ltd., Gyeonggi, Korea) and the compositions were analyzed by EDS.

### 2.4. Photocatalytic Methylene Blue Degradation

Before use as a photocatalyst, 0.2 g of each sample was distributed in 20 mL deionized water by ultrasonic treatment. The samples were then put into 200 mL of 5 mg/L methylene blue aqueous solution, and the mixture was continuously stirred by a magnetic stirrer. The photocatalytic methylene blue decomposition reaction was carried out using a UV lamp (6 W, 365 nm) at room temperature as an irradiation light source. The irradiation distance between the lamp and the sample was fixed to 10 cm. During UV irradiation, every 15 min, 2 mL of the solutions was extracted and analyzed by UV-Vis spectroscopy (G1103A, Agilent Technologies Korea Co., Ltd., Seoul, Korea) and absorption spectra were compared according to the different times of each sample.

## 3. Results and Discussion

### 3.1. Thermal Properties of Ti–Zn Complex Oxide Nanofibers

**Figure 2:**
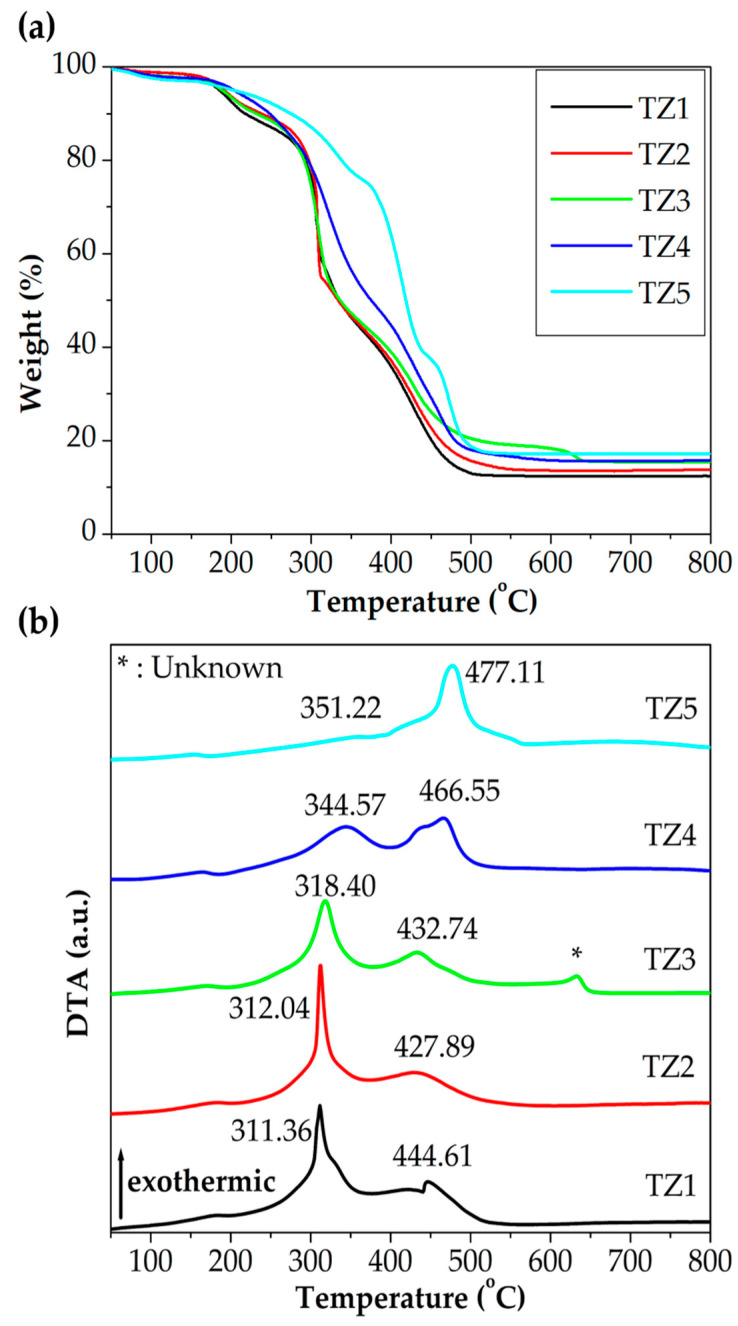
(**a**) Thermogravimetric analysis (TGA) and (**b**) differential thermal analysis (DTA) curves of as-spun Ti–Zn complex oxide nanofibers with different molar ratios of Ti and Zn in an air atmosphere (TZ1: 10:0, TZ2: 9:1, TZ3: 5:5, TZ4: 1:9 and TZ5: 0:10).

Figure 2 shows TGA and DTA curves of as-spun Ti–Zn complex oxide nanofibers with different molar ratios of Ti and Zn in an air atmosphere. After the thermal analysis, 12.4%, 13.6%, 15.7%, 15.6% and 17.2% of mass remained for TZ1, TZ2, TZ3, TZ4 and TZ5, respectively. The first exothermic reaction occurred at 311.36, 312.04, 318.40, 344.57 and 351.22 °C in TZ1, TZ2, TZ3, TZ4 and TZ5, respectively, with the zinc content increases. The loss was due to the decomposition and combustion of PVP by oxygen. The second exothermic reaction occurred at 444.61, 427.89, 432.74, 466.55 and 477.11 °C in TZ1, TZ2, TZ3, TZ4 and TZ5, respectively, with the zinc content increases. The loss was due to crystallization of Ti–Zn complex oxide. For TZ1 and TZ5, these transformations were in agreement with the literature [29,37]. For TZ2, TZ3 and TZ4, the temperature of the exothermic reaction peak changed as the ratio of Ti and Zn changed. For TZ3, a third exothermic peak at 634.44 °C was observed. A similar exothermic peak was reported in a previous report [38], but the peak was not identified.

### 3.2. Microstructural, Chemical and Crystalline Properies of Ti–Zn Complex Oxide Nanofibers

**Figure 3:**
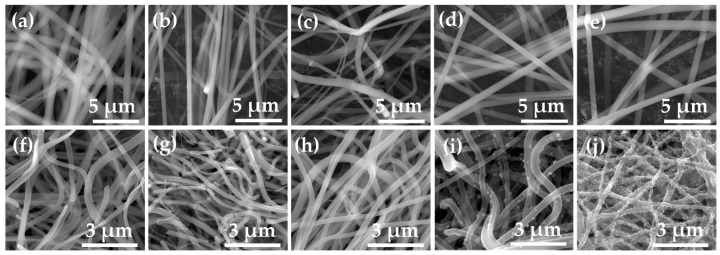
Field emission scanning electron microscope (FE-SEM) images of as-spun nanofibers with different molar ratios of Ti and Zn ((**a**): TZ1, (**b**): TZ2, (**c**): TZ3, (**d**): TZ4 and (**e**): TZ5) and annealed Ti–Zn complex oxide nanofibers with different molar ratios of Ti and Zn ((**g**): TZ1, (**h**): TZ2, (**i**): TZ3, (**j**): TZ4 and (**k**): TZ5).

**Figure 4:**
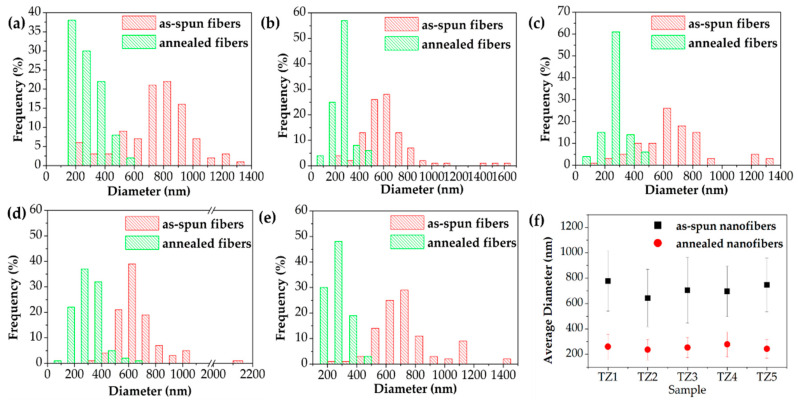
Diameter histogram of as-spun, annealed Ti–Zn complex oxide nanofibers ((**a**): TZ1, (**b**): TZ2, (**c**): TZ3, (**d**): TZ4 and (**e**): TZ5) and the average diameters (**f**) with different molar ratios of Ti and Zn.

Figure 3 and Figure 4 show FE-SEM images, the diameter histogram and the average diameter of the as-spun and annealed Ti–Zn complex oxide nanofibers. For each sample, the diameter of the nanofibers was measured at 20 intersecting points made by five vertical lines and four horizontal lines in the FE-SEM image of 40,000 magnifications, and five FE-SEM images were used to obtain a total of 100 diameter values. The average diameter and standard deviation were calculated with 100 diameter values and the histogram was plotted. Before annealing, the average diameters of TZ1, TZ2, TZ3, TZ4 and TZ5 were 777.5, 643.7, 704.8, 696.2 and 746.8 nm, respectively. After annealing, the average diameters decreased to 261.0, 237.2, 254.5, 278.6, 244.1 nm, respectively. In TZ4 and TZ5, which had a high concentration of zinc salt, spherical particles were formed on the surface of the nanofibers, and this agrees with the literature [30,39].

**Figure 5:**
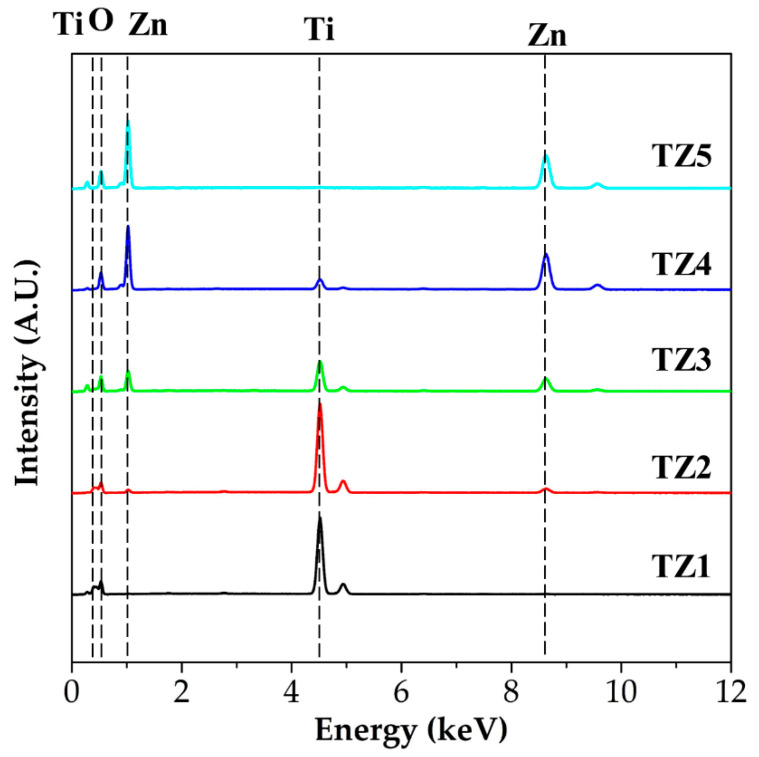
Energy-dispersive spectroscope (EDS) spectra with different molar ratios of Ti and Zn (TZ1: 10:0, TZ2: 9:1, TZ3: 5:5, TZ4: 1:9 and TZ5: 0:10).

Figure 5 shows EDS spectra of TZ1, TZ2, TZ3, TZ4 and TZ5, and Table 2 shows their chemical composition in atomic percent. In the EDS spectra, titanium peaks of 0.42 and 4.50 keV, zinc peaks of 1.02 and 8.62 keV and an oxygen peak of 0.52 keV were observed. In the order of TZ1, TZ2, TZ3 and TZ4, the Ti concentration of the annealed complex oxide nanofibers were 26.04, 31.57, 14.16, and 4.72 at%, respectively. In the order of TZ2, TZ3, TZ4 and TZ5, the Zn concentration of the annealed complex oxide nanofibers were 3.28, 13.83, 39.67, and 44.91 at%, respectively. As the Ti concentration of the precursor solution decreased and the Zn concentration increased, when comparing the ratio of Ti and Zn, the Ti concentration decreased and the Zn concentration increased. The molar ratio of the Ti and Zn of the annealed complex oxide nanofibers was found to be close to that of the metal salt of the initial electrospinning precursor solution.

**Figure 6:**
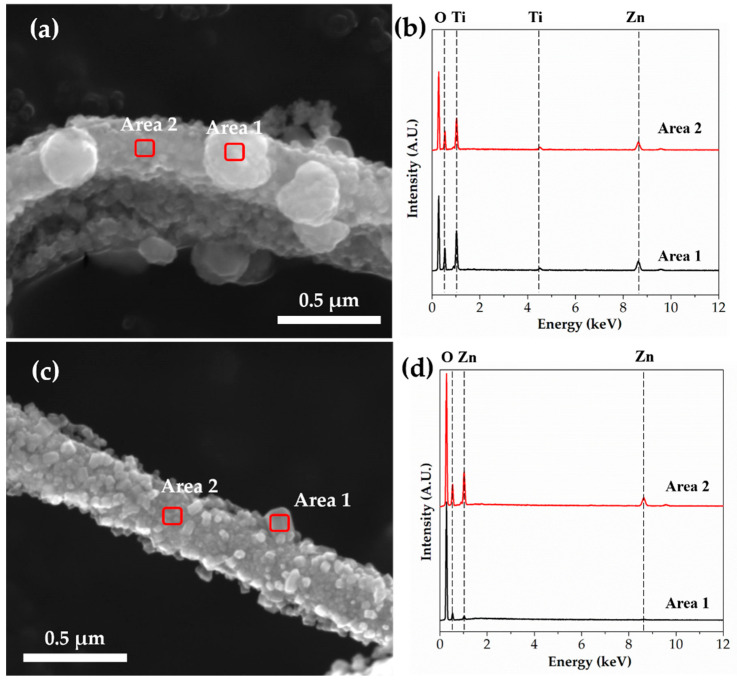
FE-SEM images and EDS spectra of (**a**,**b**) TZ4 and (**c**,**d**) TZ5 by EDS point analysis.

To observe the chemical composition of the spherical particles of TZ4 and TZ5 shown in Figure 3, EDS point analysis was conducted. Figure 6 shows their FE-SEM image and EDS spectra with different areas, and Table 3 shows their chemical composition in atomic percent. The Zn/Ti ratios in the TZ4 sample were 13.31 at area 1 and 8.24 at area 2, respectively. This reveals that spherical particles have a Zn-rich phase. Spherical particles in the TZ5 sample also have higher zinc concentration than the body of fibers. 

**Figure 7:**
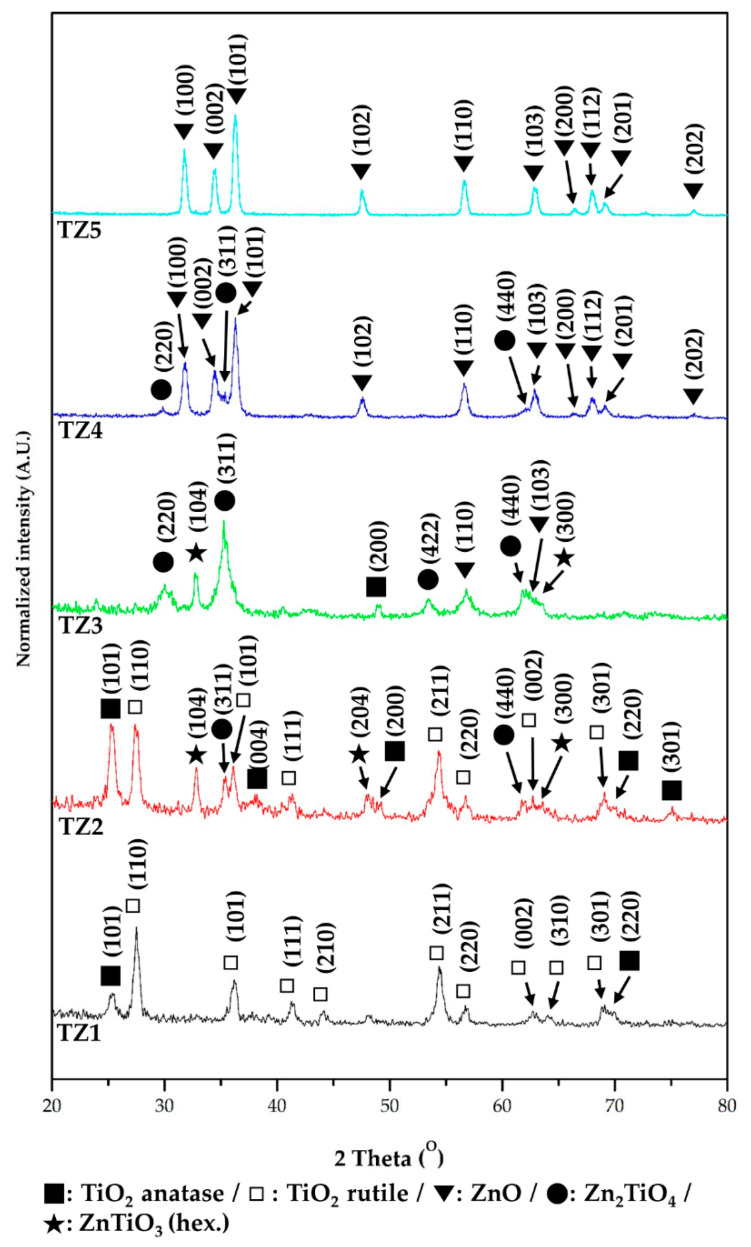
X-ray diffractometer (XRD) spectra of annealed Ti–Zn complex oxide nanofibers with different molar ratios of Ti and Zn (TZ1: 10:0, TZ2: 9:1, TZ3: 5:5, TZ4: 1:9 and TZ5: 0:10).

The crystal structure of TZ1, TZ2, TZ3, TZ4 and TZ5 with different Ti and Zn ratios was analyzed by XRD. Figure 7 shows their diffraction spectra. From the XRD pattern of the TZ1 sample, the peaks of TiO_2_ anatase and TiO_2_ rutile were identified, and this result indicates that the nanofibers prepared under the TZ1 conditions have a complex structure of dual phases of titanium dioxide. In the XRD pattern of TZ2, peaks of TiO_2_ anatase, TiO_2_ rutile, ZnTiO_3_ and Zn_2_TiO_4_ were identified, and in the XRD pattern of TZ3, peaks of Zn_2_TiO_4_, ZnTiO_3_, TiO_2_ anatase and ZnO were identified. In addition, peaks of Zn_2_TiO_4_ and ZnO were identified in the XRD pattern of TZ4. The nanofibers fabricated under TZ2, TZ3 and TZ4 conditions were mixed with Ti and Zn, and multi-metal oxide structures, namely, peaks of ZnTiO_3_ and Zn_2_TiO_4_, were observed by the reaction of TiO_2_ and ZnO. In the TZ5 sample, a peak of ZnO was identified and a single oxide structure was observed. 

### 3.3. Photocatalytic Properties of Complex Oxide Nanofibers with Different Ti and Zn Ratios

**Figure 8:**
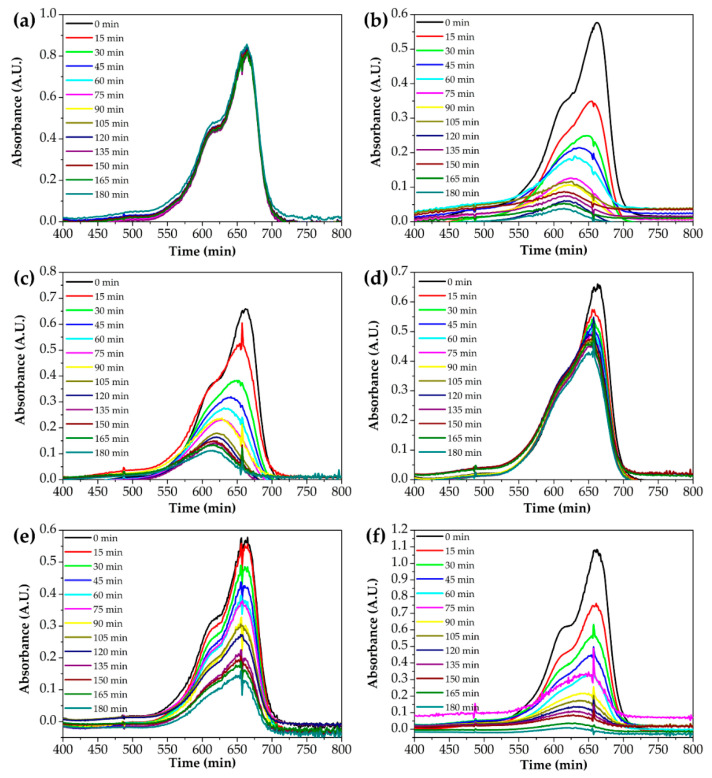
UV-Vis absorption spectra of the photocatalytic degradation of an aqueous methylene blue solution by various photocatalysts ((**a**): without catalysts, (**b**): TZ1, (**c**): TZ2, (**d**): TZ3, (**e**): TZ4 and (**f**): TZ5).

**Figure 9:**
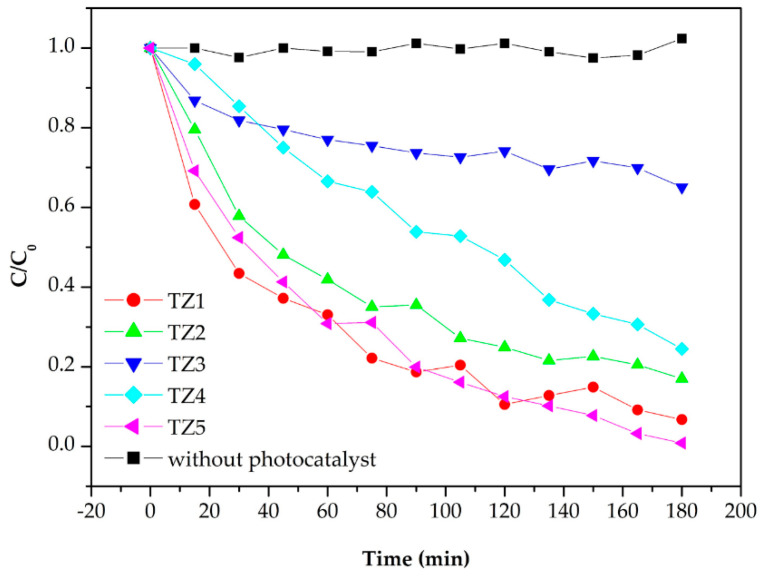
Photodegradation rate of methylene blue by various photocatalysts.

Figure 8 shows the absorption spectra to evaluate the photocatalytic performance of Ti–Zn complex oxide nanofibers observed by UV-Vis spectroscopy. The spectra show the absorbance of methylene blue aqueous solution after photocatalytic degradation without catalysts with TZ1, TZ2, TZ3, TZ4 and TZ5. Figure 9 shows that the intensity changed every 15 min. Without catalysts, the absorption intensity did not decrease. With TZ1, TZ2, TZ3, TZ4 and TZ5, the absorption intensities decreased, and the concentration decreased to 0.067, 0.167, 0.650, 0.245 and 0.008 after 180 min compared to the initial concentration of 1. These results show improved performance compared to the literature with similar experimental conditions [12]. TZ5 has a methylene blue degradation rate of 99.2%, which is about 14.2% higher than the remaining 85.0% in the literature. TZ1 had a higher photodegradation rate than TZ2, TZ3 and TZ4, which have multiple crystalline structures with Ti and Zn. The mixed structure of TZ1, which had an anatase and rutile phase, produced a better performance than the mixed structure with zinc added. Moreover, TZ5 exhibited a higher photodegradation rate than TZ1, which was due to a higher absorption rate of ZnO at 365 nm UV [40].

## 4. Conclusions

Photocatalysts are the most important technology for air pollution removal and the detoxification of organic materials. Doping and complexation are among the most used methods to improve the efficiency of photocatalysts. In this study, for use as photocatalysts, we fabricated Ti–Zn complex oxide nanofibers by a multi-nozzle electrospinning process with different molar ratios of Ti and Zn. According to the characterization and experimental results, the nanofibers had a 1-dimensional structure, and their average diameters were 237.2–278.6 nm. Multiple crystalline structures were observed and were composed of TiO_2_ anatase, TiO_2_ rutile, ZnTiO_3_, Zn_2_TiO_4_ and ZnO. We evaluated the photocatalytic performances by the degradation of methylene blue with UV irradiation. In all the nanofibers, the microstructures and phases were stable against physical damage such as ultrasonification and could be reused as photocatalytic materials by filtering and drying. A higher performance was achieved in single metal oxide structures of Ti and Zn by the multicrystalline structure of anatase and rutile, and a higher UV absorbance. In the single metal oxides of Ti and Zn, the methylene blue concentration decreased to 0.067 and 0.008, respectively, after three hours compared to the initial concentration 1, which shows the difference in photocatalytic performance in the mixed structure of TiO_2_ and ZnO, which are the most widely used photocatalyst materials. The results of this study can be used to improve the performance of photocatalysts through various process controls.

## Figures and Tables

**Table 1:** Sample names with the different Ti/Zn molar ratios of the electrospinning precursors.

Sample Name	TZ1	TZ2	TZ3	TZ4	TZ5
Ti/Zn molar ratio	10:0	9:1	5:5	1:9	0:10

**Table 2:** Chemical composition in atomic percent of TZ1, TZ2, TZ3, TZ4 and TZ5.

Sample Elements	TZ1	TZ2	TZ3	TZ4	TZ5
Ti	26.04	31.57	14.16	4.72	-
Zn	-	3.28	13.83	39.67	44.91
O	73.96	65.15	72.01	55.61	55.09

**Table 3:** Chemical composition in atomic percent of TZ4 and TZ5 by EDS point analysis.

	TZ4		TZ5	
	Area 1	Area 2	Area 1	Area 2
Zn	16.90	16.48	7.23	16.98
Ti	1.27	2.00	-	-
O	81.82	81.52	92.77	83.02
